# Polyhydroxybutyrate-Based Nanocomposites for Bone Tissue Engineering

**DOI:** 10.3390/ph14111163

**Published:** 2021-11-15

**Authors:** Anand Mohan, Madhuri Girdhar, Raj Kumar, Harshil S. Chaturvedi, Agrataben Vadhel, Pratima R. Solanki, Anil Kumar, Deepak Kumar, Narsimha Mamidi

**Affiliations:** 1 School of Bioengineering and Biosciences, Lovely Professional University, Phagwara 144411, India; madhurigirdhar007@gmail.com (M.G.); chaturvediharshil91@gmail.com (H.S.C.); agratavadhel17.ar@gmail.com (A.V.); 2 Department of Pharmaceutical Sciences, University of Michigan, Ann Arbor, MI 48105, USA; rk7410@gmail.com; 3 Special Center for Nanoscience, Jawaharlal Nehru University, New Delhi 110067, India; pratimarsolanki@gmail.com; 4 Gene Regulation Laboratory, National Institute of Immunology, New Delhi 110067, India; anilk@nii.ac.in; 5 School of Chemical Engineering and Physical Sciences, Lovely Professional University, Phagwara 144411, India; deepak.sharma99967@gmail.com; 6 Department of Chemistry and Nanotechnology, School of Engineering and Science, Tecnologico de Monterrey, Monterrey 64849, Mexico

**Keywords:** nanocomposite, tissue engineering, nano-clay, nanoblend, biomedical technology, polyhydroxy butyrate, montmorillonite

## Abstract

Bone-related diseases have been increasing worldwide, and several nanocomposites have been used to treat them. Among several nanocomposites, polyhydroxybutyrate (PHB)-based nanocomposites are widely used in drug delivery and tissue engineering due to their excellent biocompatibility and biodegradability. However, PHB use in bone tissue engineering is limited due to its inadequate physicochemical and mechanical properties. In the present work, we synthesized PHB-based nanocomposites using a nanoblend and nano-clay with modified montmorillonite (MMT) as a filler. MMT was modified using trimethyl stearyl ammonium (TMSA). Nanoblend and nano-clay were fabricated using the solvent-casting technique. Inspection of the composite structure revealed that the basal spacing of the polymeric matrix material was significantly altered depending on the loading percentage of organically modified montmorillonite (OMMT) nano-clay. The PHB/OMMT nanocomposite displayed enhanced thermal stability and upper working temperature upon heating as compared to the pristine polymer. The dispersed (OMMT) nano-clay assisted in the formation of pores on the surface of the polymer. The pore size was proportional to the weight percentage of OMMT. Further morphological analysis of these blends was carried out through FESEM. The obtained nanocomposites exhibited augmented properties over neat PHB and could have an abundance of applications in the industry and medicinal sectors. In particular, improved porosity, non-immunogenic nature, and strong biocompatibility suggest their effective application in bone tissue engineering. Thus, PHB/OMMT nanocomposites are a promising candidate for 3D organ printing, lab-on-a-chip scaffold engineering, and bone tissue engineering.

## 1. Introduction

In the last 20 years, bone-related diseases such as bone infections, bone tumors, and bone loss have been increasing globally [1]. Bone repair and regeneration is a complex process including osteoprogenitor cells proliferation and differentiation, matrix formation, and remodeling of the bone [2]. Various metal and ceramic-based materials have been developed for the treatment of bone diseases. However, metallic and ceramic materials cannot be used due to various disadvantages such as the need of surgery to remove the damaged bone [3]. In the last decade, scaffolds for bone tissue engineering have attracted attention. A scaffold is composed of cells sources such as stem or precursor cells, a matrix that can support cell processed and provide mechanical support, and growth factors or hormones [4,5]. The design and fabrication of a scaffold with suitable properties are challenging.

In the last two decades, tremendous progress has been made. However, the use of most of the new nanomaterials is limited due to toxicity and low biodegradability [6]. Recently, the interest in the field of biodegradable components has increased because of the possibility of tailoring their properties and biodegradation characteristics [7]. Polymeric nanomaterial is one of the promising materials with high biocompatibility and biodegradability compared to other inorganic nanomaterials [8]. Materials with porosity that mimic the bone architecture such as porous nanocomposites with desired mechanical properties are promising scaffolds for bone tissue engineering [9]. Among various nanocomposites, polymer-based nanocomposites are very interesting due to the fact that they can form a porous architecture and have an excellent biodegradability. Polymers also allow structural changes to tune the physicochemical properties. Hence, polymer-based nanocomposites are widely used in various tissue engineering applications such as neuro-engineering, bone engineering, and dental engineering [10,11]. Polymers combined with other nanomaterials such as nano-clay have shown more promising results in tissue engineering compared to pristine polymeric materials. In this work, we used poly(hydroxy alkanoates) (PHA) or poly(hydroxybutyrate) (PHB) polymer nanocomposite with Montmorillonite (MMT) nano-clay.

The polymer PHB shows excellent properties and great potential in bone tissue engineering. PHB-based nanocomposites have been widely described [12]. Among them, PHB is well known and widely investigated in biomedical applications due to its high biodegradability and biocompatibility. PHB is a good substitute for conventional plastics, where mechanical properties are required [13]. The thermodynamic miscibility between polymer (PHB) and nano-clays plays an important role in enhancing the properties of polymer (PHB) blends [14]. More interestingly, PHB is metabolized by the organism.

Montmorillonite (MMT) is a major ingredient of bentonite and is approved by the Food and Drug Administration of the United States (FDA) for medical use [15,16]. MMT can act as a filler in a polymeric matrix, and this has attracted the attention of scientists especially for the enhancement of the thermal and mechanical properties of native polymers [17,18]. MMT layers form stacks that are intercalated into a polymeric matrix by weak Vander Waal forces and, after expansion, become dispersed into the neat polymer [17]. MMT did not cause acute or chronic effects. Recently, MMT has gained attention due to its biocompatibility, availability, and the possibility of mixing it with a cationic agent. It has been investigated for drug and gene delivery [19]. MMT nanocomposites with biomaterials such as gelatin, collagen, silk, and chitosan were used for the fabrication of scaffolds [20,21,22,23].

In the present work, we fabricated a nanocomposite blend consisting of a PHB matrix and MMT to enhance the thermal and mechanical properties of PHB for bone tissue engineering. We investigated the effect of the ratio of MMT to PHB in the nanocomposites on the physicochemical and mechanical properties of the blend. The blending material used was montmorillonite organically modified with 25–30% (*w*/*w*) of trimethyl stearyl ammonium (OMMT) (nano-clay). The organic treatment of the clay renders the hydrophilic montmorillonite hydrophobic, thus allowing it to interface with polymeric matrices. The interfacial material is very important along with the nanofiller. Changes in the properties of the interfacial polymer may appropriately improve a nanomaterial. Long-chain alkyl ammonium is known to be successful for the synthesis and development of polymer nanocomposites with temperature stability up to 200 °C; it can be further intercalated into a polymeric matrix to obtain enhanced physical and mechanical strength of the pristine polymer. The prepared nanocomposites here described can be an excellent candidate for bone tissue engineering.

## 2. Results and Discussion

All the nanocomposites were prepared by using the solvent-casting method. Different amounts of OMMT were added to a PHB solution, and a nanocomposite film was visible after solvent evaporation. All films were dried in a hot-air oven at 60 °C and the dried films were collected. To study the effect of the composition on the physicochemical and mechanical properties of the nanocomposite films, we prepared a range of films and characterized them in detail using various advanced techniques.

### 2.1. Morphology and Surface Properties

**Figure 1:**
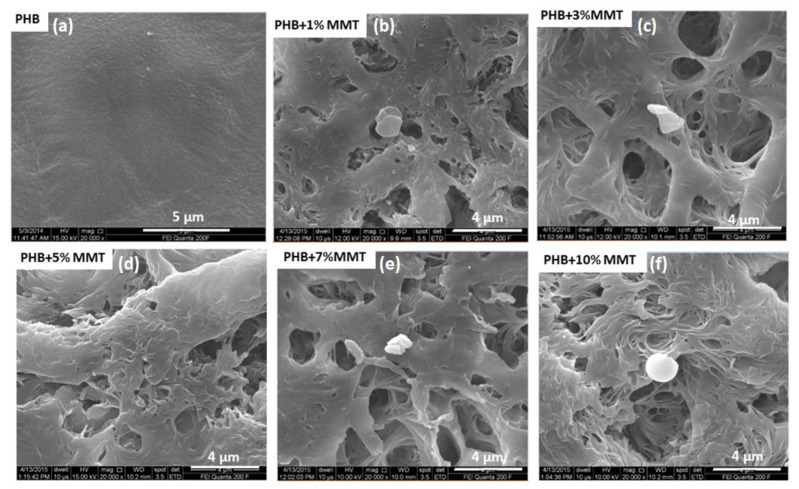
SEM images of nanocomposite films of (**a**) PHB control, (**b**) 1 wt% OMMT, (**c**) 3 wt% OMMT, (**d**) 5 wt% OMMT, (**e**) 7 wt% OMMT, and (**f**) 10 wt% OMMT.

FESEM images of the PHB/OMMT blends at different concentrations are shown in Figure 1. The small granules present on the matrix surface represent the nano-clay, whereas the bright areas indicate the matrix polymers. As observed in Figure 1, nanocomposites with different OMMT concentrations showed a mixed morphology, with the coexistence of exfoliated and intercalated clay patterns within the polymeric matrix. The observed morphology suggested a great effect on porosity of the nano-clay within the blend matrix surface. Proper shear under optimized processing temperature, rpm, and retention time, along with effective higher basal spacing and interacting groups present in the nano-clays, facilitated the easy penetration of the polymer macromolecules. The pore size was varied between 200 to 450 nm; some larger pores were also present. A higher level of porosity was observed when increasing the % of OMMT. This indicated the suitability of the current nanocomposite as a scaffold in for 3 D printing of organs. Increased porosity is known to promote homogeneous media percolation and the growth of cells over the scaffold material.

### 2.2. DSC Analysis

**Figure 2:**
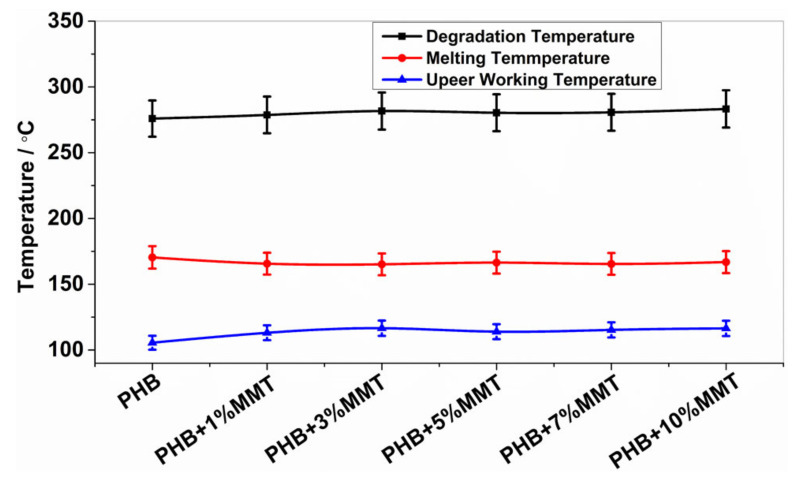
Degradation, melting, and upper working temperatures of different blends.

**Figure 3:**
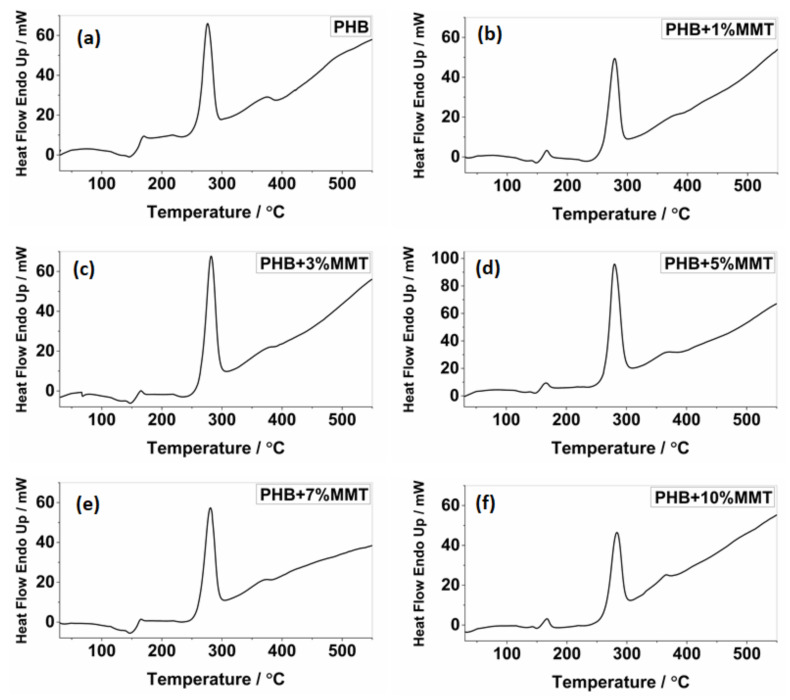
DSC thermograph showing endotherms for (**a**) pure PHB, (**b**) PHB + 1 wt% OMMT, (**c**) PHB + 3 wt% OMMT, (**d**) PHB + 5 wt% OMMT, (**e**) PHB + 7 wt% OMMT, and (**f**) PHB + 10 wt% OMMT.

The analysis of DSC thermographs (Figure 2) showed that the degradation temperature of the blends gradually increased for the blends of OMMT from 1 wt% to 10 wt%. Particularly, the 10 wt% OMMT loading film showed a degradation temperature of approximately 283.26 °C, which was higher than the degradation temperature of pristine PHB (275.92 °C). This higher degradation temperature significantly increased the working window of the 10 wt% OMMT loading film. The analysis of endothermic heat flow showed that the energy absorbed due to degradation decreased in the blended films, demonstrating that less heat flow was required at higher temperatures for degradation, as observed in the 10 wt% blended PHB film, i.e., 46.47 mJ. The degradation temperatures of the 5 wt% and 7 wt% nanocomposites did not show much variation and remained stable (Table 1). The upper working temperature of the blends significantly improved at 10 wt%, reaching 116.44 °C, which was much higher compared to that of pure PHB whose highest working temperature was 105.52 °C. Along with an increase in the degradation temperature of all blends, their melting temperature decreased, which provides a larger working window in comparison to neat PHB. This improvement in the upper working temperature associated with a large working window may help the molding of films, which could be utilized in the preparation of various scaffolds of different shapes and sizes in the field of biomedicine. The investigation revealed that the prepared nanocomposites were far more thermostable, allowing a higher range of working temperatures. In comparison to the work by Ali et al. and co-workers [15], our blend showed improved properties. All the parameters of the DSC analysis of PHB and its blends are shown in Figure 2 and Figure 3.

### 2.3. TGA Analysis

**Figure 4:**
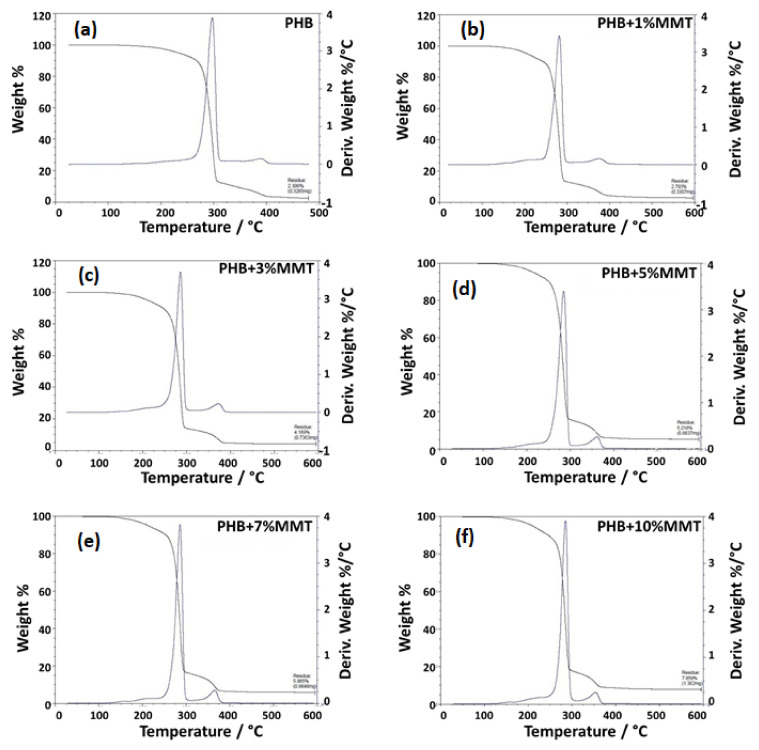
TGA thermograph showing the degradation peak of (**a**) neat PHB, (**b**) PHB + 1 wt% OMMT, (**c**) PHB + 3 wt% OMMT, (**d**) PHB + 5 wt% OMMT, (**e**) PHB + 7 wt% OMMT, and (**f**) PHB+ 10 wt% OMMT.

The TGA analysis showed that polymer/clay nanocomposites were thermally more stable than pure polymer composites [24,25]. This was because of nano-clay layers which provide superior insulation and a mass transport barrier against the decomposition of polymer volatile compounds under high-temperature conditions. This occurs because clay minerals are inorganic molecules that are stable in the temperature ranges in which pure organic polymers are susceptible to volatile conversion [26,27]. The TG thermograph showed that the weight loss of the blended films was due to degradation, which was monitored as a function of temperature. As the weight percentage of OMMT increased in the blended product, the degradation percentage of the composite decreased within a certain range. Specifically, the 7 wt% and 10 wt% blended films showed approximately 94.135% and 92.141% of degradation, respectively, as compared with pure PHB film (97.601% of degradation), as shown in the Table 1. The results for these blends are in accordance with the results reported by Zubartikudis and co-workers [27]. The analysis of the thermographs (Figure 4a–f) showed that the addition of OMMT in the polymer matrix affected the degradation percentage of the nanocomposites, which implies that OMMT was incorporated as a filler in the polymeric matrix. The incorporation of nano-clay was also found to enhance the thermal stability of the polymer. Table 1 and Figure 4 depict the wt% degradation of blended films analyzed by TGA.

### 2.4. Mechanical Properties

**Figure 5:**
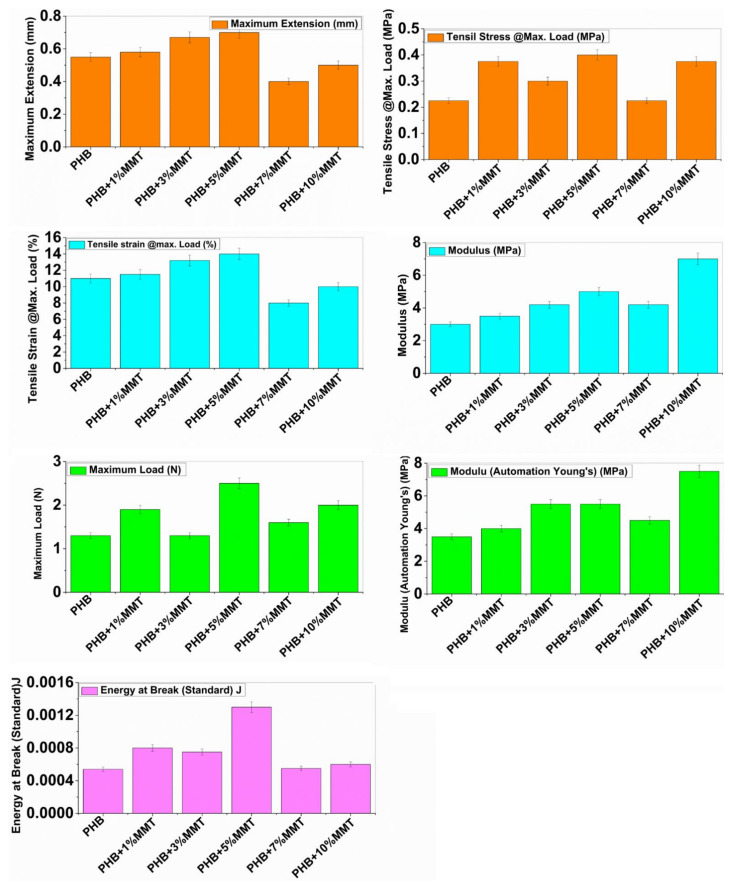
Instron mechanical testing parameters of neat PHB and different PHB blends.

The mechanical properties of neat PHB and its different blends are depicted in Figure 5. Neat PHB showed a maximum extension of 0.5525 mm, tensile stress of 0.23 MPa, tensile strain of 11.05%, modulus (Automatic) of 3.0525 MPa, maximum load of 1.35 N, modulus (automatic Young’s) of 3.4975 MPa, and energy at break of 0.00054 J. The incorporation of OMMT within the polymeric matrix resulted in intermediate properties for the blends. However, the ductility of neat PHB increased consistently with an increase in OMMT concentration from 1 wt% to 10 wt % within the PHB matrix.

The blend prepared with 10 wt% of PHB/OMMT showed an optimum increase in maximum extension of 0.668 mm, tensile stress of 0.302 MP, tensile strain of 13.304%, maximum load of 1.35N, and energy at break of 0.000764 J, and only two parameters showed the highest values, i.e., modulus (7.0825 MPa) and Young’s modulus (7.6825 MPa). For the 5 wt% loading blend, maximum readings were observed in maximum extension i.e., 0.7 mm, tensile stress i.e., 0.404 MPa, tensile strain i.e., 13.972%, energy at break i.e., 0.001324 J, and maximum load of 2.494 N. These results suggest that some degree of interaction occurred between the macromolecules of PHB and OMMT within the blend. Finely dispersed OMMT nanoparticles act as a reinforcing filler within the PHB matrix. Enhanced ductility provides better energy-absorbing capability to the PHB matrix. Overall, PHB/OMMT composite blends exhibited augmented mechanical properties compared to neat PHB. Particularly, it was observed that the 5 wt% PHB/OMMT composite presented better mechanical properties than the other blends. All mechanical parameters are shown in Figure 5.

### 2.5. Biodegradability

**Figure 6:**
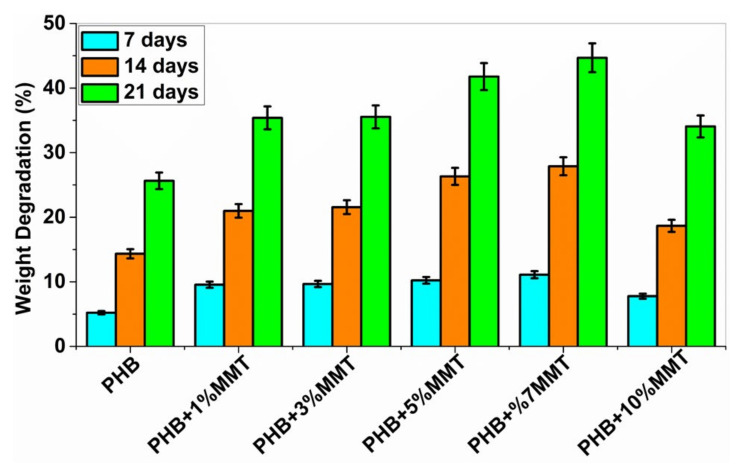
Graph showing % weight degradation of the different films by bacteria.

**Figure 7:**
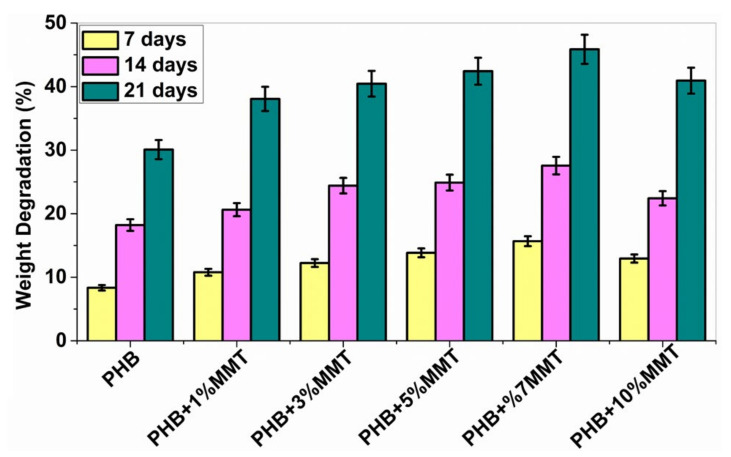
Graph showing % weight degradation of the different films by PBS.

The biodegradability of PHB polymers has been of interest to many researchers, as ester bonds of polymeric materials are hydrolyzed in the presence of CO_2_ and H_2_O. More than 300 strains of microorganisms capable of degrading PHB in vitro are widely used. Among all, the genus *Bacillus* is superior in degrading natural polymers [28]. In the current study, the degradation of PHB and PHB/OMMT nanofilms was carried out using *Bacillus subtilis* (MTCC 441) procured from IMTECH Chandigarh. A 1X PBS solution was also utilized to test the biodegradation of the nanocomposite in vitro with an incubation period of 21 days. Bacterial degradation of PHB/OMMT and pure PHB films in vitro was carried out for 7, 14, and 21 days. As the percentage of OMMT in the nanocomposite increased, the weight percentage (%) degradation also increased. The results showed that in PBS all PHB/OMMT (3 wt%, 5 wt%, 7 wt%, and 10 wt%) films had a higher rate of degradation than the pure PHB film, as shown in Figure 6 and Figure 7. These results indicated that PHB composites consisting of 5 wt% and 7 wt% blends are more biodegradable compared to neat PHB in the presence of microorganisms under natural conditions. Furthermore, the higher biodegradability and associated easy disposal allow diversified applications. The PBS assay also proved that these types of material are biodegradable, biocompatible, it can be utilized to make scaffolds, as in vivo they promote the deposition of calcium ions on injury sites and later undergo degradation within body fluids. 

### 2.6. Cell Viability Assay

**Figure 8:**
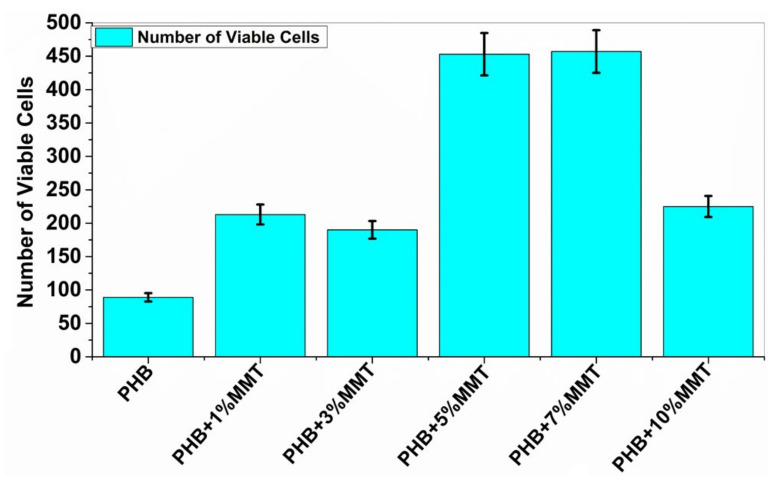
Increasing concentration of PHB + OMMT vs. no. of viable bacterial cells after 10 days of incubation.

Cell viability analysis was carried out using trypan blue dye and a hemocytometer to identify the number of viable cells. The cell viability assay analyzed the interaction of blended films with microorganisms, to evaluate the biocompatibility of microorganisms with the films. This analysis indicated that the nanocomposite films are biologically compatible as they did not negatively affect the growth of the microbial cells. The assay for all the films was carried out, and it was observed that with PHB + 7 wt% OMMT, the number of viable cells was higher as compared to that observed with the rest of the blends and neat PHB. The data reported below in Figure 8 show the number of viable cells in the presence of each film. These results confirmed the compatibility of the films with biological material and their nontoxic nature.

### 2.7. Cytotoxicity Study

**Figure 9:**
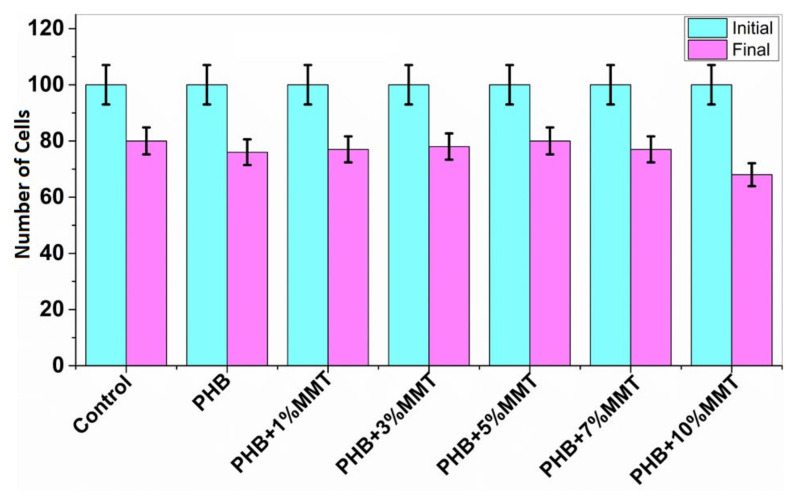
Cytotoxicity study results with the initial and final number of cells in the presence of different nanocomposites.

The MTT assay for lymphocytes was carried out in the presence of nanocomposite films at different percentages. The stained samples were observed with microscope at 10× magnification (Magnus live Olympus microscope), determining the number of non-viable cells and live cells [19]. As shown in Figure 9, the cytotoxicity results revealed that PHB films have no significant toxic effect on lymphocytes. As the weight percentage of OMMT increased in the PHB film, there was an increment in the number of live cells. We found the highest cell number for 5 wt% films in comparison to the control consisting of lymphocytes in RPMI medium. This assay is based on the reduction by mitochondrial enzymes of tetrazolium dye, which produces a purple color [29]. The results of cell viability are depicted in Figure 9.

### 2.8. Simulated Body Fluid (SBF) Results

**Figure 10:**
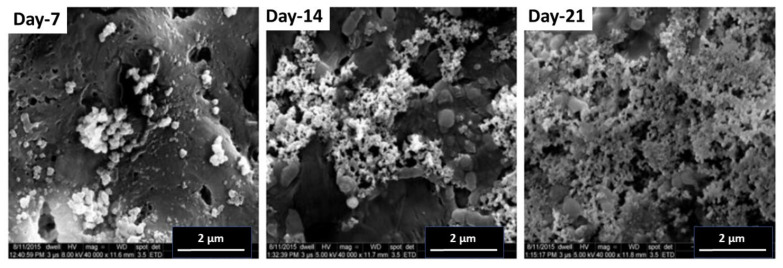
FESEM analysis after the Simulated Body Fluid Assay at different time intervals of 7, 14, and 21 days for PHB + 5 wt% OMMT.

The protocol was carried out as described in the materials and methods section to check the capability of the blends to induce osteoblast growth over the materials. SBF provides a favorable environment that is comparable to the human blood plasma and allows the deposition of calcium particles (apatite layer) over a material in vitro. This assay is based on the fact that if calcium deposition takes place over a material in vitro, then this material can induce the growth of cells over itself in vivo. It can thus be utilized in implants and other bone tissue engineering applications inside the live human body. It has been already reported that zeolites can be used both in human and in veterinary medicine as biologically active food additives (dietic additives), drugs, drug carriers, adjuvants in anticancer therapy, and antimicrobial agents. They are tolerated well in the body. Our analysis showed the deposition of an apatite layer over the test material (5 wt%) in time (7, 14, and 21 days) as shown in Figure 10. This material can thus be utilized in bone tissue engineering protocols and implants.

## 3. Material and Methods

### 3.1. Materials

Polyhydroxybutyrate (PHB) (BU396311) was purchased from the Goodfellow group of companies (ISO 9001 certified) in the granular form and was prepared by biological fermentation from renewable biological carbohydrate feedstocks. The average molecular weight of PHB was 550 kg/mol. Organically modified montmorillonite with 25–30 weight % trimethyl stearyl ammonium (OMMT) (nano-clay) was purchased from Sigma-Aldrich (product code 682608) and used as a filler for nanocomposite preparation.

### 3.2. Preparation of Nanocomposites

PHB/OMMT blends were prepared with different weight ratio percentages (1 wt%, 3 wt%, 5 wt%, 7 wt%, and 10 wt%) of OMMT in PHB by using the conventional solvent-casting method with chloroform and glass slides as the casting surface [8]. Briefly, a solution of PHB in chloroform was prepared by adding a known amount of PHB. Then, OMMT was added dropwise to the PHB solution, and the mixture was stirred for 5 h to obtain a clear and homogeneous solution. The solution was drop-casted on clean glass slides. The glass slides were kept on a clean surface in a hot-air oven at a temperature of 60 °C for 24 h. After complete drying, the formed blend film was carefully cut into pieces using a sharp knife and a scale. The slide pieces were characterized in detail.

### 3.3. Surface Morphology

The surface of the prepared nanocomposites was studied by FESEM (Field Emission Scanning Electron Microscopy). FESEM was used to analyze the interface of the nanocomposites by using a model (FEI Quanta 200F with Oxford-EDS system IE 250 × Max 80, The Netherlands) at SMITHA labs, IIT Delhi, India. A small piece of the prepared blend films was placed on a carbon tape attached to the SEM stub. Before FESEM imaging, surface conductivity was improved by coating it with gold using a vacuum sputter coater [30]. FESEM imaging was performed thoroughly by imaging at different areas of the sample.

### 3.4. Thermal Analysis

Thermogravimetric analysis (TGA) was performed using a TGA instrument (Q500 V20.10 Build 36). In total, 11.0 mg to 18.0 mg of the sample was heated from 0 to 600 °C at the rate of 20 °C/min. The analysis was carried out in a nitrogen atmosphere with a flow rate of 40 mL/min. The weight loss of the samples was recorded and plotted as the function of temperature. Differential scanning calorimetry (DSC) analysis was performed using the calorimeter STA 8000 & 8500 at the advanced center for material science at IIT Kanpur (India) to study the thermal behavior of the nanocomposite blends. Initially, the films obtained were prepared by dissecting them into squares of 2 mm × 2 mm. The squares were placed in an aluminum cell by casting and heated from 30 °C to 600 °C. The heating rate was maintained at 10 °C/min under a nitrogen atmosphere. Degradation temperature on heating, melting temperatures (T_m_), and the amount of heat flow were determined from the DSC endothermic peaks. A time lag of 2 min at 600 °C was considered, and then the samples were cooled to room temperature [31,32].

### 3.5. Mechanical Surface Morphology

The mechanical testing was performed by an Instron Microtensile Tester (Model 5848, Singapore) measuring the tensile properties, i.e., tensile stress, strain, Young’s modulus, and extension at maximum load and yield strength of the different nanocomposites. Samples with the dimensions of 30 mm × 10 mm were used for testing at room temperature, operating at 10 N and 2 kN load capacity [33,34].

### 3.6. Biodegradability

The biodegradability of the samples was studied by using bacterial cultures and 1X phosphate buffer saline (PBS). In vitro biodegradation studies were performed on different weight % of PHB/OMMT blended nanocomposites, and the results were compared with those obtained for the native PHB film. PHB degradation by many bacterial species such as *Pseudomonas, Bacillus, Azospirillum, Mycobacterium*, and *Streptomyces* species has been reported [28,35,36]. For the current study, *Bacillus subtilis* (MTCC 441) was used, and in vitro degradation was also done in PBS buffer solution. Small equal-size films were cut into pieces, pre-weighted, and inoculated in a bacterial medium and 1X PBS solution.

### 3.7. Cell Viability Assay

The viability of the bacterial cells was analyzed by inoculating the nanocomposite films into the laboratory medium (nutrient broth). The different blends of the films were individually placed in the bacterial culture and incubated at 37 °C for 10 days. A hemocytometer was used to count the viable cells after staining with trypan blue dye.

### 3.8. Cytotoxicity

The toxicity of the pure and OMMT blended PHB films was studied on isolated lymphocyte cells from human blood. Segregated lymphocytes were maintained in 96-well culture plates in RPMI 1640 medium over a period of 8 h at 37 °C in a 5% CO_2_ incubator. A hemocytometer was used for counting the lymphocytes after the MTT assay. The MTT assay (3-(4,5-dimethylthiazol-2-yl)-2,5-diphenyltetrazolium bromide) was performed to determine the toxicity effect of pure and blended PHB films in blood lymphocyte cells. An MTT stock solution was prepared by dissolving 5 mg ml^−1^ MTT in PBS; The solution was filtered through a 0.2 µm syringe filter and stored at 4 °C. Then, 100 µL of cell solution was introduced in each well along with equal sizes of pure and blended PHB films at different wt %. We then added 20 µL of MTT to each well, and the plate was incubated for a period of 4 h at 37 °C. About 100 µL of DMSO (dimethyl sulfoxide) was added, and the plate was again incubated for 1 h at 37 °C. The stained cells were counted on a hemocytometer and differentiated into viable and non-viable. The percentage of cell viability was calculated on the basis of the initial and the final count values.

### 3.9. Simulated Body Fluid (SBF) Assay

The bone-binding abilities of the biomaterials were evaluated with the SBF assay. The test is based on the formation of an apatite layer on the surface of a material implanted in the living body. Calcium phosphate ions are adsorbed by the material from the surrounding SBF fluid and stimulate the formation of a layer of apatite nuclei on the material [37,38,39,40]. The composition of SBF was NaCl 7.996 g/L, NaHCO_3_ 0.350 g/L, KCl 0.224 g/L, K_2_HPO_4_·3H_2_O 0.228 g/L, MgCl_2_·6H_2_O 0.305 g/L, 1M-HCl 40 mL/L, CaCl_2_ 0.278 g/L, Na_2_SO_4_ 0.071 g/L, (CH_2_OH)_3_ CNH_2_ 6.057 g/L; the pH was maintained at 7.4, and the temperature of the fluid at 37 °C. Nanocomposites were added to the fluid for 7, 14, and 21 days without the addition of or refreshing the SBF solution. The films were removed from the solution after the chosen times and were dried at room temperature. The formation of the apatite layer was evaluated by the FESEM technique (Model no. FEI QUANTA 200F, Netherlands operated at 15 kV) available at SMITHA LAB, Department of textile technology at the Indian Institute of Technology, New Delhi.

### 3.10. Statistical Analysis

All the experimental measurements were performed in triplicate, and the measurements are presented as mean ± standard deviation (SD). One-way analysis of variance (ANOVA) and Tukey’s post hoc tests employing Minitab 17 (Minitab, State College, PA, USA) were executed for the statistical analysis, and significance was evaluated at *p* ≤ 0.05.

## 4. Conclusions

PHB nanocomposites were successfully prepared by the incorporation of various loading wt% of OMMT using the solvent-casting method. The resulting nanocomposites exhibited augmented thermal and mechanical stability compared to the neat PHB polymer. The dispersed OMMT generated pores on the surface of the polymer. Moreover, the melting point of pristine polymers slightly increased with the incorporation of the organically modified clay. We also found that the presence of clay in the PHB matrix elevated the upper working temperature of PHB and also increased the degradation temperature upon heating. Further, a biodegradation study was performed in which blended films were exposed to *Bacillus subtilis* and PBS buffer. The results showed that as the nano-clay percentage was increased, the degradation percentage was improved when comparing the 5 wt% and 7 wt% blended films to the pure PHB film. Cytotoxicity studies showed improved cell viability along with an elevated cell proliferation percentage (16.27%) in the presence of the 5% blend, whereas all other blended polymer films showed an increase in cell proliferation. We conclude that in comparison to the PHB film, PHB/OMMT-trimethyl stearyl ammonium blended films could be a better choice for industrial use, having higher tensile and mechanical strength. Along with these properties, the resulting nanocomposite blends also induced enhanced pore formation and showed lack of toxicity towards lymphocytes. These favorable properties of the blends suggest their applicability for human cell growth and bone tissue engineering.

The advanced technology of the three-dimensional printing of organs requires suitable scaffold materials that should be highly biocompatible, non-immunogenic, and porous. Thus, the porosity of the material becomes very important, enhancing the percolation of media during 3 D printing and, thus, the growth of the cells attached to the material. The blended PHB/OMMT nanocomposite material here presented showed enhanced properties and isa potential biomaterial for 3 D printing scaffold engineering technology.

## Figures and Tables

**Table 1:** Weight percentage degradation of blends from TGA.

Name	Weight % Degraded (at 500 °C)
PHB	97.601%
PHB + 1 wt% OMMT	97.207%
PHB + 3 wt% OMMT	95.831%
PHB + 5 wt% OMMT	94.782%
PHB + 7 wt% OMMT	94.135%
PHB + 10 wt% OMMT	92.141%

## Data Availability

Data are contained within the article.
